# Olfactory impairment in psychiatric disorders: Does nasal inflammation impact disease psychophysiology?

**DOI:** 10.1038/s41398-022-02081-y

**Published:** 2022-08-05

**Authors:** Yuto Hasegawa, Minghong Ma, Akira Sawa, Andrew P. Lane, Atsushi Kamiya

**Affiliations:** 1 Department of Psychiatry and Behavioral Sciences, Johns Hopkins University School of Medicine, Baltimore, MD 21287 USA; 2 Department of Neuroscience, University of Pennsylvania Perelman School of Medicine, Philadelphia, PA 19104 USA; 3 Department of Biomedical Engineering, Johns Hopkins University School of Medicine, Baltimore, MD 21287 USA; 4 Department of Neuroscience, Johns Hopkins University School of Medicine, Baltimore, MD 21287 USA; 5 Department of Genetic Medicine, Johns Hopkins University School of Medicine, Baltimore, MD 21287 USA; 6 Department of Mental Health, Johns Hopkins University Bloomberg School of Public Health, Baltimore, MD 21287 USA; 7 Department of Pharmacology and Molecular Sciences, Johns Hopkins University School of Medicine, Baltimore, MD 21287 USA; 8 Department of Otolaryngology–Head and Neck Surgery, Johns Hopkins University School of Medicine, Baltimore, MD 21287 USA

**Keywords:** Schizophrenia, Molecular neuroscience

## Abstract

Olfactory impairments contribute to the psychopathology of mental illnesses such as schizophrenia and depression. Recent neuroscience research has shed light on the previously underappreciated olfactory neural circuits involved in regulation of higher brain functions. Although environmental factors such as air pollutants and respiratory viral infections are known to contribute to the risk for psychiatric disorders, the role of nasal inflammation in neurobehavioral outcomes and disease pathophysiology remains poorly understood. Here, we will first provide an overview of published findings on the impact of nasal inflammation in the olfactory system. We will then summarize clinical studies on olfactory impairments in schizophrenia and depression, followed by preclinical evidence on the neurobehavioral outcomes produced by olfactory dysfunction. Lastly, we will discuss the potential impact of nasal inflammation on brain development and function, as well as how we can address the role of nasal inflammation in the pathophysiological mechanisms underlying psychiatric disorders. Considering the current outbreak of Coronavirus Disease 2019 (COVID-19), which often causes nasal inflammation and serious adverse effects for olfactory function that might result in long-lasting neuropsychiatric sequelae, this line of research is particularly critical to understanding of the potential significance of nasal inflammation in the pathophysiology of psychiatric disorders.

## Introduction

Alterations in multiple sensory modalities, including auditory, visual, and olfactory processing, have been reported in patients with psychiatric disorders, such as schizophrenia and depression, and these deficits may underlie complex cognitive dysfunctions [1–9]. While pathophysiological mechanisms in the auditory and visual systems have been actively investigated [9–13], the current pandemic of Coronavirus Disease 2019 (COVID-19) highlights that chronic olfactory deficits that may impact brain function and mental health are an important and timely research topic for understanding the complex pathophysiology of psychiatric disorders.

**Fig. 1:**
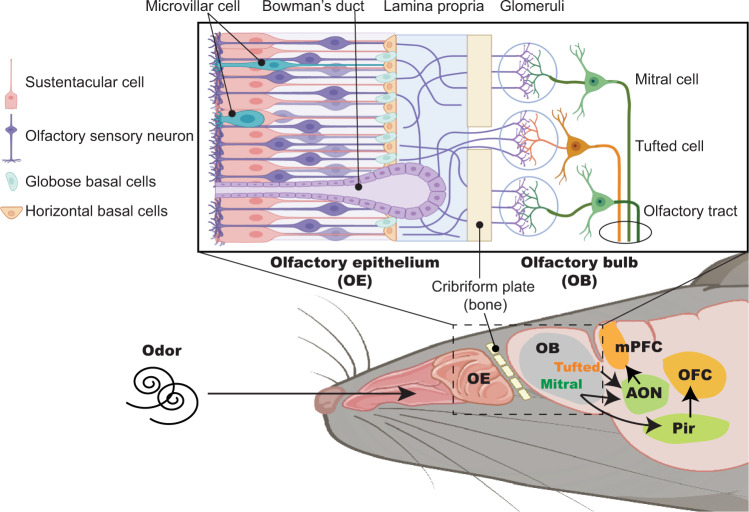
Anatomy of olfactory epithelium and neural connection with the olfactory system and higher cerebral cortex in the rodent. Schematic representation shows tissue and cellular structure of the olfactory epithelium (OE) and the projection of olfactory sensory neurons (OSNs) into the olfactory bulb (OB). In the OE, OSNs are produced from basal cells and project to the glomerular layer of the OB where OSNs make synaptic connections with OB neurons, including mitral and tufted cells. The mitral/tufted cells subsequently relay olfactory sensory information to primary olfactory cortical regions, including the anterior olfactory nucleus (AON) and the piriform cortex (Pir). Recent research has uncovered neural circuit connections between the olfactory system and prefrontal regions such as the medial prefrontal cortex (mPFC) and orbitofrontal cortex (OFC), which regulate higher brain functions (i.e., the OB-AON-mPFC and OB-Pir-OFC pathways).

Evolutionarily, the olfactory system is a crucial sensory modality, essential for survival behaviors and behavioral adaptation upon detecting odor cues [14, 15]. Odor information is initially perceived by olfactory sensory neurons (OSNs) in the olfactory epithelium (OE) inside the nasal cavity and is transmitted to the olfactory system, which is comprised of the olfactory bulb (OB) and primary olfactory cortices, including the anterior olfactory nucleus (AON) and the piriform cortex (Pir) [16, 17]. Recent neuroscience research has uncovered neural connections between the olfactory system and higher cerebral cortices, including the medial prefrontal cortex (mPFC) and orbitofrontal cortex (OFC), which are associated with higher brain functions such as cognition, memory, motivation, and emotion [18–22] (Fig. 1).

Accumulating evidence suggests that olfactory impairments are involved in the pathology of Alzheimer’s disease and Parkinson’s disease [23, 24]. In addition to neurodegenerative diseases, there is also compelling evidence that olfactory impairments are implicated in psychiatric disorders [4, 5, 25–28]. Impairment of odor discrimination associated with schizophrenia was initially reported in 1988 [29]. Since then, many studies reported that olfactory performance, demonstrated through measures of odor identification, odor discrimination, and/or odor detection, is impaired in patients with schizophrenia, psychosis, and depression [4, 7, 26, 30–45]. More recently, pathological changes in the olfactory system, including a reduction in OB volume, aberrant functional connectivity among brain regions critical for olfactory processing, and neuronal and molecular changes in the OE have also been found in patients with schizophrenia and psychosis [41, 46–49]. OB volume loss has also been observed in patients with depression [50]. Olfactory deficits are associated with negative symptoms, impaired social and cognitive functioning, and depressive symptoms [31–33, 36, 43, 45, 50–56]. Furthermore, olfactory dysfunction may be a significant pathological hallmark in the early stages of disease progression that include first episode psychosis [26, 42, 57, 58]. Although these results support the pathological implication of olfactory impairments in psychiatric disorders, how olfactory dysfunction affects the neural mechanisms underpinning higher brain functions remains poorly understood.

There is a large body of clinical evidence indicating that inflammatory processes are involved in the pathophysiology of schizophrenia and depression [59, 60]. Complementary findings from preclinical studies highlight aberrant systemic and brain immune systems as potential mechanisms underlying neuroinflammation that lead to behavioral outcomes relevant to these psychiatric disorders [61–63]. This notion is supported by epidemiological findings that environmental factors such as air pollutants and viral infections contribute to the risk for psychiatric diseases including schizophrenia and depression [64–68], perhaps at least in part via nasal inflammatory mechanisms. However, much less is known about the specific pathological role of OE inflammation for neurobehavioral consequences and disease pathophysiology.

In this article, we will first give an overview of published findings on how nasal inflammation impacts the olfactory system. We will also summarize clinical evidence on olfactory impairment in psychiatric disorders, with a particular focus on schizophrenia and depression. We will then provide an overview of preclinical studies on the neurobehavioral outcomes produced by olfactory dysfunction. Finally, we will discuss the potential impact of OE inflammation on brain development and function, as well as in disease-associated mechanisms, which may contribute to understanding the importance of OE inflammation in the pathophysiology of psychiatric diseases.

## Nasal inflammaton impacting olfactory system

### Anatomy of the OE and OB

The OE is located at the dorsal and posterior portion of the nasal cavity, variably extending inferiorly along the nasal septum and turbinates [69]. Histologically, the pseudostratified OE is thicker than the respiratory epithelium and lacks motile cilia. The apical sustentacular cells surround the cell bodies and dendritic projections of mature OSNs. Underneath this layer are immature neurons and progenitor cells including horizontal basal cells (HBCs). The half-life of OSNs is about 90 days, and the OE has a remarkable capacity for regeneration, with normal turnover of OSNs through globose basal cell proliferation and differentiation [70, 71]. As a result of severe OE damage, quiescent HBCs become active and can differentiate into not only OSNs, but also non-neuronal cells, such as sustentacular cells, globose basal cells, and Bowman’s gland cells, regenerating the entire neuroepithelium [72]. Bipolar OSNs have sensory cilia that extend from dendritic knobs into the nasal cavity. These OSNs have axonal projections that cross through the foramina of the cribriform plate of the ethmoid bone to reach the glomeruli of the OB. Newly regenerated OSNs express a given odorant receptor and precisely project onto discrete glomeruli of the OB that contain axons expressing the same odorant receptor [72]. The OB projection neurons (mitral and tufted cells) then relay olfactory sensory information to primary olfactory cortices such as the AON and Pir [16, 17] (Fig. 1).

### Nasal inflammation impacting the OE and OB

OE inflammation has been extensively studied in chronic rhinosinusitis, which is a common heterogeneous inflammatory condition of unknown origin. Regardless of whether it results from an external trigger (e.g., environmental allergens, irritants, air pollutants, or microbes) or an underlying intrinsic immune dysregulation, sinonasal inflammation causes symptoms of nasal congestion, drainage, and, in many cases, a diminished sense of smell. Most likely, a reduction in airflow plays an important role in olfactory loss as there is decreased conduction of odorants to the olfactory cleft. However, there is also a significant sensorineural component to olfactory loss that is not completely understood. This is at least in part because chronic inflammation damages the olfactory neuroepithelium and inhibits its regeneration [73, 74].

The cellular and molecular mechanisms underlying air pollutant-induced nasal inflammation have begun to be investigated, mainly with a focus on the effect of particulate matter (PM) [75]. For instance, PM-treated human nasal epithelial cells or tissue samples exhibited an elevation of pro-inflammatory molecules (such as tumor necrosis factor (TNF), interleukin 1β (IL-1β), interleukin 6 (IL-6), and interleukin 8 (IL-8)), a transition of macrophages to a pro-inflammatory state, and disrupted epithelial barrier function due to a reduction of tight-junction proteins [76–79]. Consistent with these findings, an accumulation of immune cells such as macrophages, neutrophils, and eosinophils, an elevated expression of IL-1β, interleukin 13 (IL-13), and eotaxin-1, and reduced expression of tight-junction proteins, such as claudin-1 and epithelial cadherin, are observed in the sinonasal tissue of the PM-exposed mice [80].

Respiratory viral infections, such as influenza virus and coronavirus, induce olfactory inflammation [81, 82]. Preclinical studies also suggest that some of these infections induce central nervous system (CNS) inflammation or have access to the CNS via an olfactory route [83–86]. In addition, recent studies on COVID-19, which is caused by severe acute respiratory syndrome coronavirus 2 (SARS-CoV-2), have illustrated that it may lead to nasal inflammation and olfactory dysfunction [87, 88]. The molecular and cellular mechanisms underlying the effect of SARS-CoV-2 infection on the olfactory system and olfactory sensory loss have recently begun to be investigated [89, 90]. The lack of expression of viral entry factors, such as Angiotensin Converting Enzyme-2 (ACE2), suggests that OSNs may not be direct targets for SARS-CoV-2 [91, 92]. Instead, sustentacular cells, HBCs, and Bowman’s gland cells, which do express viral entry proteins [91, 92], may be a reservoir of viral replication, which could result in OE cellular damage and inflammation, leading to disruption of OSN function [93]. However, preclinical studies demonstrate that when nasally administered, the S1 subunit of the SARS-CoV-2 spike protein spreads to multiple brain areas with the highest entry into the OB [94], indicating OSNs possibly being the first route for viral invasion to the brains [95, 96].

It is conceivable that OE inflammation impacts central olfactory neural structures. Both chronic rhinosinusitis as well as neurodegenerative and psychiatric diseases are found to involve OB volume loss [46, 50, 97–99]. Furthermore, recent evidence suggests that chronic rhinosinusitis is associated with an increased risk of psychiatric symptoms such as depression, anxiety, and cognitive dysfunction [100–102].

In order to investigate how nasal inflammation impacts OE function, a genetic mouse model of inducible olfactory inflammation (IOI) has been developed [103]. In this Tet-on system, by crossing Tet-response element (TRE)-Tumor Necrosis Factor (TNF) mice with the cyp2g1-reverse tetracycline transactivator (rtTA) line, one can drive expression of TNF specifically in OE sustentacular cells, inducing chronic and local OE inflammation in a temporally controlled manner [103]. Using this mouse model, we have previously reported on the critical role of HBCs as direct participants in the progression of chronic OE inflammation and have identified a concomitant Nuclear Factor κB (NF-κB)-mediated functional switch away from OSN reproduction [74]. In another mouse model, Hasegawa-Ishii et al. demonstrated that chronic nasal inflammation induced by intranasal lipopolysaccharide (LPS) administration induces loss of OSNs and results in neuroinflammation, gross atrophy of the OB, and loss of synaptic contacts onto tufted cells, more severe than mitral cells [104]. Glial activation and pro-inflammatory cytokine expression were proposed by the authors to contribute to OB atrophy. Since OSN axons contribute to the olfactory nerve layer (ONL) and glomerular layer (GL), but not the external plexiform layer (EPL), shrinkage of the superficial layers of the OB and recovery from nasal inflammation-induced atrophy may not be entirely explained by OSN loss. The EPL is comprised of secondary dendrites of projection neurons, including mitral and tufted cells, which synapse with granule cell dendrites. EPL gliosis induced by the intranasal LPS administration model is implicated as a contributing factor to atrophy [104, 105]. However, evidence also suggests that the lack of sensory inputs due to OSN loss results in EPL shrinkage both in the LPS model and in odor deprivation models [106–108] relating to retraction of dendrites of OB projection neurons. The impact of the loss of odor-signaling inputs may not be limited to the OB, as the AON also shrinks in response to odor deprivation [109] and semilunar cells of the Pir undergo apoptosis [110, 111]. Further study is required to examine whether nasal inflammation-induced OB circuitry changes affect cortical areas receiving inputs from mitral and tufted cells.

## Olfactory impairment in psychiatric disorders

### Olfactory pathology in schizophrenia

Olfactory impairments have gained growing interest in psychotic disorders, such as schizophrenia [4, 5, 25, 48]. Substantial evidence from neuropsychological studies reveals several facets of olfactory dysfunction, such as impaired odor identification and odor discrimination, in individuals with schizophrenia, early onset psychosis, or high risk for psychosis [4, 26, 30–32, 35–40, 42, 43, 45, 57, 112]. In contrast, findings related to odor detection threshold are mixed and incongruent in patients with schizophrenia [33, 113–116]. Previous studies have shown that these olfactory deficits are largely independent from the effects of cigarette smoking and psychiatric medications [30, 117–119]. However, a recent meta-analysis has shown that heavy smoking is paradoxically associated with smaller deficits in olfactory function in patients with schizophrenia [26]. This study also revealed that previous studies in which patients were on a regimen of first-generation antipsychotics showed greater olfactory deficits than those in which patients were treated with second-generation antipsychotics [26]. It is also worth noting that although some patients with schizophrenia experience olfactory hallucinations, several studies have demonstrated no association between olfactory hallucinations and olfactory impairments [120–122], suggesting that these phenotypes may be mediated by different neural mechanisms.

Accumulating evidence does indicate that various olfactory deficits are associated with negative symptoms and impaired social and cognitive function in patients with schizophrenia and early psychosis [31–33, 36, 43, 45, 51, 54, 56, 112]. For instance, many studies have consistently reported that impaired odor identification is associated with negative symptoms and social dysfunction in patients with schizophrenia [32, 33, 36, 51, 54, 56]. Some studies have also revealed an association between odor identification deficits and cognitive impairments [56, 123]. Furthermore, impaired odor discrimination has recently been shown to be associated with negative symptoms and cognitive impairments in patients with schizophrenia [42, 45]. Several studies have reported that impaired odor identification is associated with negative symptoms in patients with early psychosis as well [31, 43, 112, 124, 125]. A few studies have also revealed an association between reduced olfactory discrimination and negative symptoms or cognitive impairments in first-episode psychosis [43, 126]. Furthermore, two studies have shown that odor identification deficits are associated with negative symptoms in high risk individuals [127, 128]. One of these studies also showed an association between impaired olfactory identification and cognitive impairments in this population [128]. A prospective cohort study reported that although there were no differences in olfactory identification at baseline or follow-up between high risk individuals who transitioned to psychosis and those who did not, individuals with poor functional outcomes showed significantly lower baseline olfactory identification than those with good outcomes [40]. The authors concluded that impaired olfactory identification may be a useful marker to distinguish high risk individuals who may experience a poor functional outcome, regardless of transition status.

Neuroimaging studies reveal structural and functional abnormalities in various brain regions involved in olfactory processing in schizophrenia [41, 46, 48]. These brain regions include the OB and primary olfactory cortices, which have an underappreciated neural connectivity with higher brain regions such as the mPFC and OFC which regulate higher-order information processing [18–22]. In addition, recent studies have revealed reduced OB volume in patients with first episode psychosis compared to healthy controls [49]. Interestingly, physiological and brain abnormalities in the olfactory system are also observed in young high-risk individuals who developed schizophrenia, as well as their first degree relatives [26, 57, 58]. This finding suggests that assessing olfactory functioning may provide an early pathological sign and biological marker in the disease progression, including during the prodromal stages before the onset of schizophrenia, which typically occur in early adulthood.

While the pathological implication of olfactory impairment for schizophrenia has been extensively studied, the specific role of nasal inflammation in the developmental course of olfactory dysfunction is underexamined. The OE is the peripheral region of the olfactory system in the nasal cavity, where OSNs are directly exposed to the environmental stimuli [17]. Several studies have reported neuronal and molecular changes in the OE of patients with schizophrenia. In a pioneering study using immunohistochemical approaches, Arnold and colleagues reported a reduction in p75 nerve growth factor receptor (p75NGFR)-positive basal cells and an increase in growth-associated protein 43 (GAP43)-positive immature OSNs in the postmortem OE tissue of patients with schizophrenia, suggesting altered developmental composition of the OSNs [47]. More recently, using the OE collected by nasal biopsy from schizophrenia patients, microarray expression studies have revealed that differential expression of molecules in the small-mothers-against-decapentaplegic (SMAD) pathway, which is involved in inflammatory processes regulated by transforming growth factor-β (TGF-β) [129], is associated with cognitive impairments [130]. In this study, the number of cigarettes smoked per day was evaluated as an independent variable in linear regression analysis, which revealed no significant effect of smoking on gene expression in the OE samples, suggesting that smoking did not account for these trends. Furthermore, recent RNA sequencing-based molecular expression studies reported that certain molecular pathways involved in the immune/inflammatory system, such as the NF-κB signaling pathway, are altered in olfactory neuronal cells produced from the OE, and that these changes are correlated with OB volume in patients with first episode psychosis [49]. While the exact molecular and cellular mechanisms behind these abnormalities are unknown, these pathological phenotypes may be produced by OE inflammation. Human OE-derived cell/tissue models could be a promising system to address these questions [131].

Respiratory viral infections (e.g., influenza virus and coronavirus) induce olfactory inflammation [81, 82]. Preclinical studies suggest that some of these infections induce CNS inflammation or have access to the CNS via an olfactory route [83–86]. Epidemiological evidence indicates that not only maternal infection, but also childhood and adulthood infection, increase the risk of developing schizophrenia [64, 132–139]. Recent retrospective cohort studies showed a bidirectional association between diagnoses of COVID-19 and psychiatric disorders, including psychosis and depression [140]. Other large cohort studies have reported high psychiatric complications in patients with COVID-19, including psychosis and cognitive dysfunctions [141]. Overall, although a causal link between nasal viral infections and schizophrenia risk remains elusive, the nasal inflammation produced by respiratory viral infections may contribute to olfactory impairments in the psychopathology of schizophrenia.

In addition, recent prospective cohort studies support air pollution in childhood as an environmental factor for increased risk for schizophrenia [65, 67]. For instance, a ten-year follow-up study in a Denmark cohort reported that childhood nitrogen dioxide (NO_2_) exposure is associated with an increased risk of developing schizophrenia [142]. Another study following a UK cohort for 18 years from birth reveals an association between NOx exposure and increased psychotic experience during adolescence [143]. A one year follow-up study found that exposure to air pollution during childhood is particularly associated with impairment of attention, memory, and learning abilities [144]. Given that adolescence is a vulnerable period in which environmental stimuli can alter PFC maturation, as well as a critical time for the emergence of onset of schizophrenia in adulthood [145], the potential impact of adolescent exposure to air pollutants and viral infection on brain maturation and longitudinal schizophrenia risk also warrant further investigation.

Altogether, this clinical evidence supports the intriguing hypothesis set out in this review that nasal inflammation induced by developmental exposure to these environmental factors may alter neural circuit maturation in the olfactory system and perhaps in even higher cerebral cortex areas involved in the regulation of higher brain functions that are relevant to schizophrenia.

### Olfactory pathology in depression

Olfactory impairment is also implicated in depression. One recent study systematically analyzed a reciprocal relationship between olfaction and depression that has been reported in previous studies [7, 50, 52, 53, 55, 120, 146–152]. The study demonstrated that olfactory function, including olfactory threshold, discrimination, and identification, is impaired in depressive patients, and, further, that patients with olfactory impairments experience worse depressive symptoms with greater severity of smell loss [7]. Correspondingly, evidence also suggests that the degree of olfactory impairment varies depending on duration and course of depression [44]. Thus, it is of interest to investigate whether nasal inflammation may contribute to the pathophysiology of depression via olfactory dysfunction.

Respiratory viral infections and air pollutants are potential environmental risk factors not only for schizophrenia and psychosis, but also for depression. For instance, a population-based study showed that people with a previous influenza infection had an increased risk of developing depression [153]. Similarly, COVID-19 infections are associated with increased rates of newly recognized depression [140]. Previous studies have also demonstrated the pathological implication of air pollutants (i.e., particulate matter 2.5 [PM_2.5_]) in depressive patients [66, 67]. In the context of gene-environment interaction, PM_2.5_ exposure is involved in altered cortical neural circuit networks in patients with depression [68].

The pathological implication of nasal inflammation is also supported by evidence that chronic rhinosinusitis is associated with an increased risk of depression [101, 102]. Allergic rhinitis is induced by IgE-mediated reactions to inhaled allergens, which produce an inflammatory reaction in the nasal mucosa [154]. Epidemiological evidence suggests that a history of seasonal allergies confers a heightened risk of depression [155]. Furthermore, a population-based prospective case-control study showed that allergic rhinitis during adolescence increases the risk of depression [156]. These results indicate that nasal inflammation produced by various factors (e.g., viral infection, air pollutants, and chronic and allergic rhinitis) may contribute to a greater risk for depression.

Studies have reported reduction of OB volume in patients with major depressive disorder, as well as negative correlation between OB volume and depression scores [50]. Olfactory and emotional processing are regulated by shared neural circuits and brain regions such as the amygdala that receive sensory information from the OB [27], supporting the notion that olfactory impairments may be involved in the pathophysiology of depression [28, 157]. Nevertheless, given that these results are produced from cross-sectional studies with relatively small cohorts, further studies with larger sample sizes and longitudinal designs are required. Histochemical and molecular phenotyping of the OE in depressive patients is also areas of future investigation.

## Olfactory dysfunction impacting neurobehevioral phenotypes

### Neurobehavioral outcomes produced by olfactory dysfunction

Behavioral changes caused by olfactory dysfunction are observed in multiple rodent models (Table 1). Surgical removal of the OB in mice and rats, namely olfactory bulbectomy, results in hyperactivity, altered sleep patterns, aberrant stress-induced coping responses, and abnormalities in various cognitive functions such as spatial memory performance, recognition memory, motivational behavior, and fear learning [158–164]. Given that these phenotypes include non-odor-guided behaviors (e.g., fear learning), olfactory bulbectomy does not solely impair olfaction, but also affects higher-order cognitive processing regulated by the primary olfactory cortices and downstream brain regions that include the hippocampus, amygdala, mPFC, and OFC. Indeed, optogenetic stimulation of OSNs induces rhythmic activity in the OB and mPFC and disruption of olfactory input impairs the neural activity of the mPFC [20].

**Table 1:** Rodent models of olfactory dysfunction.

Model	Behavior	Biology	Reference
Inducible olfactory inflammation (IOI) model	Impaired sociability Impaired preference for social novelty Dampen consumatory pleasure	Chronic and local OE inflammation Increased pro-inflammatory cytokine in OB	Chen et al. 2019 [74] Hasegawa et al. 2021 [172] Lane et al. 2010 [103]
Air pollutants exposure (2-etthyl-1-hexanol, PM2.5, ozone)	Impaired social recognition memory Impaired learning and memory Altered coping responses	Inflammatory cell infiltration in OE Microglia number increase in OB Loss of OSN Elevated pro-inflammatory cytokine in the HPC Aberrant morphology of the HPC neurons	Miyake et al. 2016 [176] Fonken et al. 2011 [178] Guevara-Guzman et al. 2009 [179]
Intranasal virus infection (VSV: vesicular stomatitis virus)	No studies	Inflammatory cell infiltration in OE and OB Microglia activation in OB	Moseman et al. 2020 [177]
Chronic rhinosinusitis model (Intranasal ovoalbumin, pollen, Triton X-100, LPS injection)	Reduced social interaction Anxiety-like behavior	Loss of OSN Gross atrophy of OB Microglia and astrocyte activation in OB Increased pro-inflammatory cytokines in OB	Tonelli et al. 2009 [180] Hasegawa-Ishii et al. 2017 [105] Hasegawa-Ishii et al. 2019 [104] Kim et al. 2019 [181] Hasegawa-Ishii et al. 2020 [182]
Olfactory bulbectomy	Impaired recognition memory Impaired spatial memory Impaired fear learning Impaired motivational behaviors Altered coping response Hyper activity Altered sleep pattern	Increased 5-HT2 receptor binding Decreased BDNF expression in HPC Decreased cell proliferation in DG Hypotrophic neurons in piriform cortex Aberrant neuronal arborization in CA1 Impaired long-term potentiation(LTP) Increased TNF-a and IL-6 in CC and HPC Increased caspase-3 activity in CC and HPC	Gurevich et al. 1993 [158] Song and Leonard. 2005 [159] Moriguchi et al. 2006 [160] Hendriksen et al. 2012 [161] Rinwa and Kumar. 2013 [162] Flores et al. 2014 [163] Hendriksen et al. 2015 [164]
*Cnga2* genetic deletion	Social behavior deficit Impaired spatial memory Anxiety-like behavior	Aberrant dendritic morphologies in HPC	Chen et al. 2014 [165] Matsuo et al. 2015 [166] Xie et al. 2016 [167]
M71 odorant receptor overexpression	Anxiety-like behavior	Elevated plasma corticosterone Odor-evoked glomerular activity alteration Octanal-induced seizure	Glinka et al. 2012 [168] Nguyen and Ryba. 2012 [169] Roland et al. 2016 [170]
*Rag-1* genetic deletion	Anxiety-like behavior Impaired odor-sensing	Disorganized glomeruli in OB	Rattazzi et al. 2015 [171]
Methimazole-induced loss of OSNs	Increased freezing behavior	Reduced 4-Hz oscillation in plPFC Local inflammatory response in OE	Chen et al. 2017 [73] Moberly et al. 2018 [20]
Tetrodotoxin infusion into OB	Increased freezing behavior	Reduced 4-Hz oscillation in plPFC	Moberly et al. 2018 [20]

*OB* Olfactory bulb, *OE* Olfactory epithelium, *OSN* Olfactory sensory neurons, *CC* Cerebral cortex, *HPC* Hippocampus, *DG* Dentate gyrus, *plPFC* Prelimbic prefrontal cortex.

Behavioral outcomes have also been elicited by genetic inhibition of olfactory function. Genetic deletion of *cyclic nucleotide gated channel subunit alpha 2* (*Cnga2*), which is essential for regulating odorant signal transduction, resulted in impaired spatial memory and social behaviors, as well as anxiety-like behavior [165–167]. OSN-specific overexpression of M71 odorant receptors produced aberrant odor-evoked neural activity, leading to octanal-induced seizures and anxiety-like phenotypes [168–170]. Genetic deletion of the recombination activation gene (*Rag-1*), which is expressed not only in the lymphoid cells but also in OSNs, caused disorganized glomeruli structure in the OB, impaired odor-sensing, and anxiety-like behavior [171]. Furthermore, a pharmacological olfactory lesion induced by an intraperitoneal injection of methimazole and an infusion of tetrodotoxin into the OB resulted in increased freezing behaviors during retrieval of conditioned fear [20, 73]. Differences in observed behavioral phenotypes in these rodent models may be explained by different experimental approaches used to impair olfactory function. Although these results reinforce the importance of the underappreciated olfactory pathways informing regulation of higher brain function, the underlying molecular and neural circuit mechanisms remain obscure.

It should be noted that rodents largely depend on olfaction for sensing the external world—more than humans do—and that their performance in specific behavioral phenotypes such as social behaviors, heavily depends on olfaction. By examining the behavioral impact of manipulating specific olfactory-prefrontal neural circuits such as the OB-AON-mPFC and OB-Pir-OFC pathways, rodent models may help us to identify neural substrates involved in olfactory modulation of higher brain functions. In particular, it is of interest to examine the roles of olfactory-prefrontal circuits in the modulation of behavioral outcomes in positive valence systems, social processes, and cognitive systems: areas in which disturbances are associated with olfactory impairments in psychiatric disorders [31–33, 36, 43, 45, 51, 54, 56].

### OE inflammation as an entry point causing pathology of psychiatric disorders

In order to examine whether nasal inflammation-induced disturbance in the peripheral olfactory system indeed causes behavioral alterations of neuropsychiatric relevance, we have recently investigated the adverse effects of chronic and local OE inflammation on behavioral consequences using the aforementioned inducible olfactory inflammation (IOI) mouse model [74, 172]. While no abnormalities in locomotion and anxiety phenotype were observed, IOI mice exhibited impairment of sociability and preference for social novelty in the three-chamber social interaction test, suggesting that chronic OE inflammation impairs social behaviors that highly rely on olfactory cues in rodents [173]. The sucrose preference test was also used to characterize a loss of consummatory pleasure, which is a component of anhedonia [174]. IOI mice exhibited a lower preference for the sucrose solution compared to controls, suggesting that OE inflammation may dampen consummatory pleasure. Considering that olfactory deficits are correlated with social and cognitive abnormalities, as well as negative symptoms in psychosis [32, 36, 45, 54, 56, 123], relevant behavioral domains in this mouse model warrant future investigation.

In addition, rodent models of exposure to environmental risk factors for psychosis such as air pollutants and microbial infections displayed OE inflammation and OB volume loss [175–177]. Adolescent mice chronically exposed to air pollutants showed learning and memory deficits [178]. Chronic exposure to ozone has also been shown to impair olfactory perception and social recognition memory in rats [179]. The importance of OE inflammation’s impact on higher brain function is also supported by rodent models of chronic rhinosinusitis. These models produce olfactory impairments, OSN loss, OB volume reduction, OB-mPFC circuit disruption, and behavioral abnormalities including altered social behaviors [104, 105, 180–182]. These results, together with the epidemiological evidence described above, suggest that the OE may be an entry point for the deleterious effect of inflammatory environmental factors such as air pollutants and viral infection on the CNS.

**Fig. 2:**
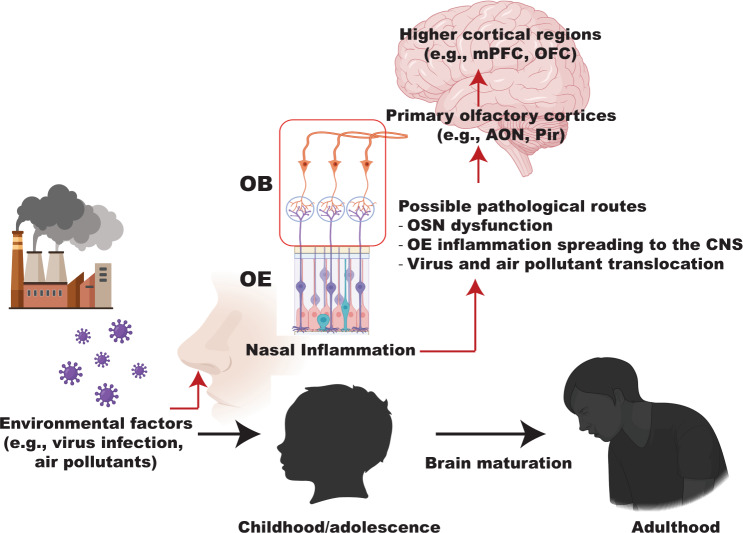
Adverse effects of nasal inflammation on brain development and function. Schematic representation shows the olfactory epithelium (OE) as an entry point for developmental exposure to environmental inflammatory factors (such as air pollutants and viral infection) and their adverse effects on the central nervous system, initially through the olfactory bulb (OB). OE inflammation may alter maturation of the olfactory system and its functional connectivity to the distal brain regions involved in regulation of higher brain function relevant to psychiatric disorders, and it may have these effects via multiple routes from the OE. mPFC, medial prefrontal cortex; OFC Orbitofrontal cortex, AON Anterior olfactory nucleus, Pir Piriform cortex.

The question arises of how local OE inflammation contributes to neural dysfunction underlying behavioral phenotypes relevant to psychiatric disorders. There are several possibilities to be explored for the mechanistic routes of OE inflammation impacting the CNS: 1) OSN dysfunction induced by OE inflammation resulting in abnormalities in the structural or functional composition of the OB; 2) OE inflammation spreading to the CNS through the OB; and/or 3) direct translocation of virus and air pollutants to the olfactory system. These changes may contribute to disturbance of olfactory-prefrontal circuits, leading to neurobehavioral consequences relevant to psychiatric symptoms (Fig. 2).

As described above, olfactory deficits are observed early in disease progression, including during the prodromal stages [26, 43, 57, 58]. Adolescence is a critical period for both PFC maturation and the prodromal process of psychiatric disorders [145, 183, 184] and the peripheral olfactory system displays a high degree of plasticity [185–187]. Thus, it is worth investigating whether OE inflammation-induced molecular and neural alterations during developmental periods such as adolescence may lead to long-lasting behavioral abnormalities in adulthood. The IOI mouse model may be useful in investigating this possibility, as it allows us to temporally control OE-specific expression of TNF, provoking local and chronic OE inflammation [74, 103, 172]. One can envision that OSN dysfunction may be a major contributory factor mediating the adverse neurobehavioral effects of OE inflammation. Crossing TRE-inward rectifier potassium channel transgenic mice with the olfactory marker protein (Omp) promoter-driven tTA line (*Omp-tTA;TRE-Kir2.1*) [188–190] will allow us to determine whether non-inflammatory OSN dysfunction negatively impacts olfactory-prefrontal circuits and resultant behaviors. Given that genetic risk factors play an important role in the etiological complexities of psychiatric diseases, it is also crucial to explore the convergent mechanisms of genetic risk factors and nasal inflammation.

## Conclusions and perspectives

In the past decade, multiple lines of evidence from clinical studies and neuroscience research have shed light on the underappreciated olfactory pathway for regulation of higher brain function and its implications for the pathophysiology of psychiatric disorders such as schizophrenia and depression in a cross-disease manner. As growing evidence suggests that inflammatory processes play a role in disease pathophysiology, it is important to explore if and how OE inflammation induces molecular and neuronal alterations in the OE that provoke impairment in olfactory circuit-mediated brain systems, leading to neurobehavioral consequences relevant to these disease conditions.

This area of research is particularly critical when we consider the current outbreak of COVID-19, which may increase the risk of psychiatric disorders via nasal inflammation. However, it should be noted that the psychological stress produced by social isolation and restriction may also be involved in the increased risk of psychiatric disorders in patients with COVID-19. Although this variable is difficult to address in clinical research, it is testable in preclinical studies. By using rodent models of social isolation and SARS-CoV-2 infection, we can explore how social isolation may have a convergent effect with SARS-CoV-2 infection on brain function, which may include disturbances involved in the psychopathology of psychiatric disorders. Psychiatric consequences of COVID-19 should also be longitudinally monitored, and future preclinical investigations are needed to characterize the pathological effect of SARS-CoV-2 infection on brain function.

In summary, the findings discussed above suggest that nasal inflammation impairs the peripheral olfactory system and may affect olfactory-higher brain circuits, leading to neurobehavioral abnormalities. Further research into the molecular, cellular, and circuit mechanisms underlying the effects of nasal inflammation on brain function is crucial in addressing how OE inflammation contributes to the adverse effects of environmental factors such as air pollutants and viral infection on the central nervous system, potentially leading to neuropsychopathology relevant to psychiatric disorders.
